# Multivariate analysis reveals significant diuron-related changes in the soil composition of different Brazilian regions

**DOI:** 10.1038/s41598-019-44405-x

**Published:** 2019-05-27

**Authors:** Paulo Sérgio Fernandes das Chagas, Matheus de Freitas Souza, Jeferson Luiz Dallabona Dombroski, Rubem Silvério de Oliveira Junior, Glauber Henrique de Sousa Nunes, Gustavo Antônio Mendes Pereira, Tatiane Severo Silva, Ana Beatriz Rocha de Jesus Passos, José Barbosa dos Santos, Daniel Valadão Silva

**Affiliations:** 1 Universidade Federal Rural do Semi-Árido, Mossoró, Rio Grande do Norte Brazil; 2 Universidade Estadual de Maringá, Maringá, Paraná Brazil; 3 Universidade Federal de Viçosa, Viçosa, Minas Gerais Brazil; 4 Universidade Federal dos Vales do Jequitinhonha e Mucuri, Diamantina, Minas Gerais Brazil

**Keywords:** Environmental impact, Chemical biology

## Abstract

Sorption and desorption determine the amount of an herbicide in soil solution. Therefore, knowledge of the sorption and desorption coefficients in different soils is an essential factor to estimate the potential for environmental contamination by herbicides. We evaluated the feasibility of multivariate and linear discriminant analyses to predict the sorption and desorption capacity of a soil for diuron, one of the most used herbicides on sugarcane plantations. The adsorptive capacity in twenty-seven Brazilian soil samples was estimated using the sorption constant (Kfs) and desorption constant (Kfd) obtained by the Freundlich isotherms. The regression model was created from the sorbed and nonsorbed concentrations of diuron in soils. Ultra-performance liquid chromatography was applied to quantify the diuron concentrations. The multivariate analysis separated the soils into four groups considering the similarity of the following attributes: pH, organic matter, clay, and base saturation. The groups showed a similar pattern of sorption and desorption for diuron: Lom-Lclay: low sorption (5.9 ± 1.2) and high desorption (10.9 ± 0.6); Lclay: low sorption (7.5 ± 1.1) and high desorption (11.4 ± 1.3); Hom-Hclay: high sorption (11.2 ± 1.2) and low desorption (13.8 ± 1.2); HpH-Hclay: high sorption (10.1 ± 1.1) and medium desorption (11.5 ± 1.4). Linear discriminant analysis of these soil attributes was used to classify other soils described in the literature with adsorption capacity. This analysis was able to identify soils with high and low sorption using the pH, organic matter, clay, and base saturation, demonstrating the enormous potential of the technique to group soils with different contamination risks for subterranean waters. Sugarcane crops in northeastern Brazil showed a higher pollution risk through the leaching of diuron. Multivariate analysis revealed significant diuron-related changes in the soil composition of different Brazilian regions; therefore, this statistical analysis can be used to improve understanding of herbicide behavior in soils.

## Introduction

The application of pesticides in modern agriculture has allowed production systems to minimize losses caused by pests, diseases, and weeds^1^. Among agrochemicals used in agriculture, herbicides represent 54% of the total consumption, occupying the prominent position in the sales market^2^. Most of the pesticides applied in Brazil are used in areas with sugar cane cultivation. In 2017, the area planted with this crop was equivalent to 8.73 million hectares, and this production is destined to supply the sugarcane industry (CONAB, 2018). Among the agrochemicals applied during the productive cycle of sugarcane, herbicides are often used as a management tool. This crop has a long critical weed period of prevention, requiring frequent applications or use of herbicides with a longer residual period^3^.

An herbicide that is commonly used in areas cultivated with sugarcane is diuron [N-(3,4-dichlorophenyl)-N, N-dimethylurea]. This herbicide belongs to the chemical group of substituted ureas, with a water solubility of 42 mg L^−1^ at 25 °C and a Kow (octanol/water) partition coefficient of 589^4^. When applied during pre-emergence, diuron is very efficient for controlling dicotyledonous and some monocotyledons species, increasing the frequency of its application to sugarcane crops^5^.

Despite the advantages of using diuron in weed control, its application can cause environmental contamination^6^. The half-life of diuron in soils can reach 30 to 150 days^7,8^ (moderate persistent), but a half-life of 330 days for this herbicide has been reported in the literature^9^. Additionally, in sandy soil, this herbicide demonstrates high mobility, and after the occurrence of rainfall or irrigation, this herbicide can contaminate groundwater resources^10^. For example, researchers have already identified the presence of diuron in rivers and groundwater in different Brazilian states^11,12^. However, the environmental contamination caused by herbicides can be avoided when the interactions between the soil and herbicide are known.

Among soil-herbicide interactions, sorption and desorption define the amount available in the soil solution and, consequently, the mobility of this pesticide in the environment^13^. The sorption is defined as the soil capacity to adsorb an herbicide by chemical and physical interactions such as van der Waals forces, hydrogen bonds, ionic bonds, hydrophobic bonds, charge transfer complexes, electrostatic interactions, and covalent bonds. The reverse process characterizing the return of the sorbed herbicide to the soil solution is denoted desorption. The intensity of the sorption and desorption of an herbicide to soil depends on the physical and chemical properties of the herbicide molecule and the soil^14^. Therefore, the diversity of physical and chemical attributes of Brazilian soils makes it challenging to predict the behavior of diuron. Thereby, studies involving large numbers of soil samples from different regions are necessary to evaluate the behavior of this herbicide.

Brazilian soils are classified in 13 groups (Fig. S2). However, three soils classes (oxisols, ultisols and cambisols) occur more frequently^15^. This classification is not sufficient to estimate the sorption and desorption of an herbicide. For instance, oxisols exhibit considerable variability in their sorption capacity for diuron, rendering ineffective the use of this classification in herbicide recommendations. For a safety recommendation concerning an herbicide, it is necessary to have a knowledge of the soil attributes, such as the organic matter and clay content, pH, cation exchange capacity, and soil texture^16,17^. Several studies have previously demonstrated the sorption and desorption of diuron in many soils, and in some cases, the application of diuron has led to a higher risk of groundwater contamination due to the low sorption to soil^18,19^. Nevertheless, this information has not been compilated to monitor areas with a high risk of pollution due to exposure to this herbicide.

Some studies have previously demonstrated the successful application of the multivariate analysis to group soils based on their physical and chemical properties, resulting in groups with a similar adsorption capacity of trace elements^20^ and heavy metals^21^ and soils with a similar erosion potential^22^. These groups can allow the elaboration of reference values that will help to monitor areas and conditions of risk due to the adoption of agricultural practices. If these grouped soils demonstrate similar sorption and desorption capacities for an herbicide, it is possible to use a function discriminant to evaluate the contamination risk of other soils based on their physical and chemical attributes. However, it is necessary to select the best variables that will permit the grouping and provide the discriminant function with a high hit percentage.

In this study, we sought to explore the use of multivariate statistics as a tool to help monitor the risk potential of diuron based on its sorption and desorption in different Brazilian soils. For this purpose, we constructed three hypotheses: (1) What soil attributes should be used for the cluster and discriminant analysis? (2) Do soils collected from sugarcane-producing states show a similar pattern of sorption and desorption when grouped due to the similarity of their physical-chemical attributes? (3) Are there Brazilian regions with higher contamination risks due to the application of diuron? (4) Will the linear discriminant analysis classify other soils with higher and lower sorption and desorption capacities? The results demonstrated that a linear model from discriminant analysis could be construed to identify agricultural areas with a high risk of contamination by diuron. Additionally, the study revealed Brazilian regions with a higher risk of groundwater contamination due to diuron application.

## Material and Methods

The experiments were conducted at the Weed Management Laboratory of the Federal Rural University of the Semi-Arid. The equilibrium time, sorption and desorption tests were performed using the ultrahigh-performance liquid chromatography (UHPLC) technique in triplicate.

### Soil collection

Soil samples were collected from different Brazilian states (Table S1 and Fig. S1). The samples were collected from the 0–20 cm layer in areas that were free of herbicide application. The number of samples for each state varied based on three criteria: (1) The soils were collected in regions of Brazil with historical and/or emerging sugarcane cultivation to identify areas subject to diuron application. (2) The number of samples in each region varied according to the heterogeneity of the soils to maximize the breadth of the sorption and desorption values of diuron in Brazil. (3) The soils should be free of diuron residues to allow equilibrium time, sorption, and desorption procedures to be performed according to the Organization for Economic Co-operation and Development (2000)^23^ guidelines. The absence of diuron in the collected soils was confirmed by chromatography tests using control (soil) and fortified samples (soil+ a known concentration of diuron); the details are shown in Fig. S2.

Twenty-seven soil samples were collected in 9 Brazilian states. The soils were air dried, sieved using a 2 mm mesh and characterized chemically (Table S2) and physically (Table S3) according to the methodology proposed by EMBRAPA (2013)^15^. After characterization of the physical-chemical attributes, the soils were given a new identification (S1, S2, S3… S27). This procedure ensured that cluster analysis only considered the characteristics of each soil, grouping them by similarity among attributes, regardless of the state in which the soil was collected. The cited procedure resulted in the creation of new groups containing soils from different and/or the same states.

### Reagents

The experiment was conducted using analytical grade reagents and HPLC. The diuron used had a purity of 99.6%. A stock solution of diuron was prepared at a concentration of 1000 mg L^−1^ in acetonitrile. The working solutions were prepared by diluting the stock solution in 10 mmol L^−1^ CaCl_2_ as the diluent.

### Analytical method

Diuron quantification was performed using the ultrahigh-performance liquid chromatography system, which consisted of a Shimadzu DAD UHPLC detector (nexera model X2 SPD-M30A) and 75 mm long × 2 mm internal diameter Shimadzu C18 stainless steel column (Shim-pack XR- ODS III (1.6 μm). The chromatographic conditions consisted of a mobile phase of water (acidified with 0.1% HCOOH) and acetonitrile at a proportion of 3:7, injection volume of 3 μL, flow rate of 0.3 mL min^−1^ and detection wavelength of 245 nm.

### Determination of the equilibrium time for sorption

The equilibrium time required for the sorption and desorption of diuron in soils was performed by the “batch equilibrium” method^23^. To Falcon tubes containing 2 grams of soil was added 10 ml of a solution of the herbicide at a concentration of 1 mg L^−1^, prepared in 10 mmol L^−1^ CaCl_2_. Subsequently, the tubes were agitated vertically at different time intervals (10, 20, 30, 60, 120, 240, 480, 720, 960 and 1440 minutes) at 25 °C ± 2 °C. After stirring, the samples were centrifuged at 2260 g for seven minutes. The supernatant was removed and filtered using a 0.22 μm PVDF membrane directly into 1.5 ml vials. Subsequently, the samples contained in the vials were analyzed by ultrahigh-efficiency liquid chromatography (UHPLC) to quantify the concentration of diuron.

### Sorption equilibrium

The sorption of diuron in soils was evaluated using working solutions prepared from a stock solution at concentrations of 0.2, 0.4, 0.73, 1.5, 3, 6 and 12 mg L^−1^ of the herbicide in 10 mmol L^−1^ of a CaCl_2_ solution. The isotherm points were prepared using a concentration of 0.73 mg L^−1^, which corresponds to the recommended dose for clay soils. Falcon tubes containing 2 g of soil were filled with 10 mL of the solution containing the herbicide in 10 mmol L^−1^ of CaCl_2_ solution. The concentration of solution added to the tubes varied according to the previously defined concentrations. Then, the tubes were shaken vertically at 25 °C ± 2 °C for the equilibration time for each soil sample. After stirring, the samples were centrifuged at 2260 g for seven minutes. The supernatant was then removed and filtered using a 0.22 μm PVDF membrane directly into 1.5-mLvials. The concentration of diuron in the samples contained in the vials was quantified by ultrahigh-efficiency chromatographic analysis (UHPLC).

The herbicide adsorbed in the soil (Cs) was quantified in mg kg^−1^ based on the difference between the concentration of standard solution initially added to the soil (Cp) in mg L^−1^ and the concentration found in the equilibrium solution (Ce) in mg L^−1^. The values of Ce and Cs were adjusted by the Freundlich equation (Cs = Kf_s_ Ce^1/n^) for interpretation of the sorption process.

### Desorption equilibrium

The desorption tests were performed with the tubes used in the sorption experiment. After removal of all supernatants in the sorption test, 10 mL of herbicide free 10 mmol L^−1^ CaCl_2_ solution was added to these tubes. Subsequently, the tubes were closed and shaken in a vortex mixer for 10 seconds. Vertical stirring was again performed for the same period applied in the equilibration time trials at a temperature of 25 ± 2 °C. A portion of the supernatant was then collected and filtered using a 0.22 μm PVDF membrane directly into 1.5-mL vials and analyzed by UHPLC.

Quantification (mg kg^−1^) of the concentration of herbicide that remained adsorbed to the soil after desorption was determined based on the difference between the concentration of herbicide adsorbed in the soil before the desorption steps and the concentration in the analyzed solution. The obtained values were adjusted using the Freundlich equation (Cs = Kf_d_ Ce^1/n^).

### Linearity of the method, analytical curve and selectivity

The highest absorption of diuron was detected at wavelengths 211 and 251 nm (Fig. S2). The selectivity of the method was evaluated by analyzing the supernatants, resulting from agitation of the soil with 10 mL of 10 mmol L^−1^ CaCl_2_ solution without and with diuron (Fig. S2). This procedure was also used to confirm the absence of diuron in the soil. Samples without diuron showed no peaks at wavelengths of 211 and 251 nm. Linearity was determined using 10 concentrations of diuron in triplicate, ranging from 0 to 20 mg L^−1^ in 10 mmol L^−1^ CaCl_2_ (Fig. S3).

### Data analysis

The results obtained in the analyses of the chemical and physical attributes (Tables S2 and S3) of the soils were evaluated by Pearson’s correlation, cluster analysis (CA), factorial analysis (FA) and discriminant analysis (DA). The data were standardized with a mean = 0 and variance = 1 to avoid the interference of units of soil attribute measurements in the cluster and factorial analysis. The variables used in CA, FA, and DA were determined using the Pearson correlation (p ≤ 0.05), opting for those with a lower correlation and that usually affect the sorption and desorption of diuron. The cluster analysis was carried out by adopting the Euclidean distance and Ward method to link the cases.

The cut distance in CA was defined as 5.8, and this value was determined using the method proposed by Mahalanobis (1930)^24^. The factors derived in the FA were rotated by the Varimax method. Only factorial loads with values greater than 0.65 were considered significant. Variables with values of commonality less than 0.50 were considered to lack a sufficient explanation^20^. Validation of the classification of the groups was performed by the DA, applying the Mahalanobis distance and the probability proportional to the size of the group. The linear function provided by DA was used to group other soils with known sorption and sorption coefficients. The pH, MO, V, and clay of these soils was used to group the soil by the classification functions (highest value obtained in each group). Eighteen soils were selected from data that were previously published in the literature^25–29^.

After AC, AF, and AD, the averages for Kf_s_ and Kf_d_ were calculated within each group. The confidence interval was determined to measure the dispersion of the Kf_s_ and Kf_d_ values within each group. The averages and confidence intervals were used to compare the behavior of diuron within the groups. The potential leaching of diuron was calculated using the GUS index. Two values (maximum and minimum) for the half-life of diuron in the soil based on previous research^7,8^ were used to estimate the Groundwater Ubiquity Score (GUS index). The half-life values were 15 and 150 days. The GUS index (1) was calculated using the pesticide half-life (from the literature) and Koc (from laboratory data). The Koc was obtained by dividing the value of Kfs by the organic carbon content of the soil.

1 $$GUS\,index={\rm{loLom}}-{\rm{Lclay}}0\,({\rm{half}}-{\rm{life}})\times [4\,-{\rm{loLom}}-{\rm{Lclay}}0\,({\rm{Koc}})$$

All the statistical analyses were performed by computation in the environment R (R Development Core Team, 2005). Graphs were elaborated using Sigmaplot^®^ Version 12.0.

## Results

### Factorial, Cluster and discriminant analysis

The values for pH, organic matter (OM), saturation of bases (*V*) and clay presented a high correlation with at least one of the other soil attributes (Table 1). This finding allowed the exclusion of other soil attributes in further analyses (cluster and discriminant). The four selected soil attributes by Pearson’s correlation were used in the factorial analysis, generating three new factors (Factorial 1 to Factorial 3) capable of explaining 93.7% of the total variability of the twenty-seven soils (Table 2). Factorial 1 allowed the estimation of the influence of base saturation and clay on the 27 soils used. The Factorial 2 and Factorial 3 factors were formed by the pH and MO (Table 2). The obtained percentage in the factorial analysis indicate that the attributes pH, OM, V, and clay could detect 90% of all similarities among soils in the cluster analysis (CA); thereby, CA could be performed using these four attributes.

**Table 1:** Coefficients of simple linear correlation (r) between attributes of the 27 soils.

	pH	^a^MO	P	K	Ca^+2^	Mg^+2^	Al^+3^	H + Al	^b^CTC	^c^V	^d^m	^e^Ar-Sil	Clay
pH	1.00	0.11	0.34	0.26	0.47	0.30	−0.44	−0.46	−0.25	0.58	−0.71	−0.02	0.10
^a^MO	0.11	1.00	0.05	0.39	0.32	0.30	−0.28	0.48	0.81*	0.17	−0.36	−0.44	0.42
P	0.34	0.05	1.00	0.42	0.57	0.29	−0.17	−0.30	0.42	0.56	−0.20	−0.15	0.72*
K	0.26	0.39	0.42	1.00	0.87*	0.54	−0.27	−0.20	0.56	0.72	−0.34	−0.64*	0.73*
Ca^+2^	0.47	0.32	0.57*	0.87*	1.00	0.54	−0.31	−0.34	0.56	0.90*	−0.40	−0.59	0.71*
Mg^+2^	0.30	0.30	0.29	0.54	0.54	1.00	−0.21	−0.13	0.45	0.62*	−0.28	−0.50	0.47
Al^+3^	−0.44	−0.28	−0.17	−0.27	−0.31	−0.21	1.00	0.10	0.06	−0.35	0.98*	0.24	−0.21
H + Al	−0.46	0.48	−0.30	−0.20	−0.34	−0.13	0.10	1.00	0.10	−0.59*	0.08	−0.11	0.02
^b^CTC	−0.25	0.31	0.42	0.56	0.56	0.45	0.06	0.10	1.00	0.78*	0.04	−0.42	0.49
^c^V	0.58*	0.17	0.56	0.72*	0.90*	0.62*	−0.35	−0.59*	0.38	1.00	−0.44	−0.43	0.52
^d^m	−0.71*	−0.36	−0.20	−0.34	−0.40	−0.28	0.98*	0.08	0.04	−0.44	1.00	0.28	−0.26
^e^Ar-sil	−0.02	−0.44	−0.15	−0.64*	−0.59	−0.50	0.24	−0.11	−0.42	−0.43	0.28	1.00	−0.90*
Clay	0.10	0.42	0.72*	0.73*	0.71*	0.47	−0.21	0.02	0.49	0.52	−0.26	−0.90*	1.00

^a^Organic carbon. ^b^Effective cation exchange capacity. ^c^Base Saturation. ^d^Saturation by aluminum. ^e^Sand +silt. *Significant at 5%.

**Table 2:** Factorial axes extracted for soil attributes and respective factor loads, eigenvalues, total and accumulated variance, and commonality.

Variable	Factorial axis^a^			Communality
	Factorial 1	Factorial 2	Factorial 3	
	Factorial loads^b^			
pH	−0.65	−0.67	0.32	0.94
Organic matter	−0.53	0.68	0.55	0.99
Base saturation	−0.86	−0.30	−0.23	0.89
Clay	−0.74	0.41	−0.43	0.94
Eigenvalues	1.99	1.10	0.67	—
Total variance (%)	49.66	27.51	16.79	—
Accumulated variance (%)	49.66	77.18	93.97	—

^a^Factorial axes rotated by the Varimax method. ^b^Factorial loads ≥|0,65| were considered significant for interpretation purposes.

**Figure 1:**
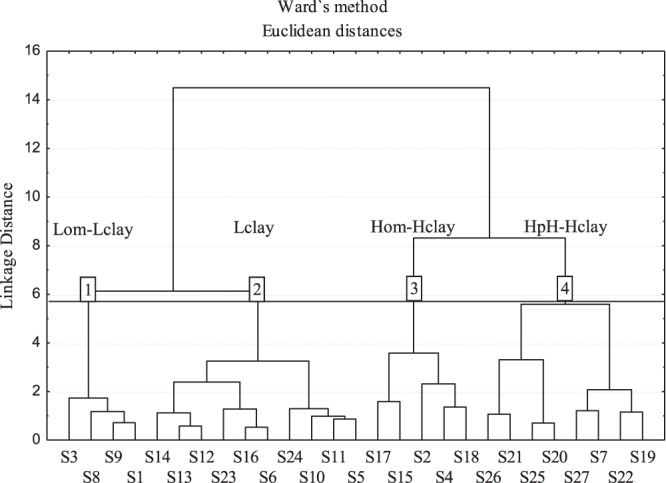
Dendrogram of the soil samples in four groups, produced by the Ward method from the Euclidean distances, obtained as a function of the variables pH, organic matter, sum by bases and clay.

The cluster analysis (Ward method) using the attributes pH, MO, V, and clay separated the 27 soils into four groups: Lom-Lclay (Low organic matter and clay content); Lclay (Low clay content); Hom-Hclay (high organic matter and clay content); HpH-Hclay (high pH and clay content). The group with the highest number of samples was Lclay, followed by HpH-Hclay, Hom-Hclay, and Lom-Lclay (Fig. 1). The four soils that were grouped in the Lom-Lclay were collected in the Rio Grande do Norte state. This was the only group containing soil samples from one state (Rio Grande do Norte). The other groups presented soils from different Brazilian regions (Fig. 1). This new distribution of soils would not necessarily group the soils in terms of the state of origin, but rather according to their physicochemical characteristics. The Lom-Lclay grouping with soils of the Rio Grande do Norte state was based solely on the similarities between attributes.

After the grouping, a discriminant analysis was applied to create four linear functions (one for each soil group), where each attribute (pH, MO, V, and clay) received a weight. These weights varied among the groups (Table 3). Discriminant analysis showed that 100% of the soil samples were correctly allocated to their respective groups (Table 3). The maximum hit index of the AD (100%) indicated that all the soil groupings presented a high degree of similarity for the attributes pH, MO, V, and clay.

**Table 3:** Classification functions of the groups defined by the *cluster* analysis and validated by the discriminant analysis.

Groups	Classification Functions	^b^Total samples	^c^Hit (%)
^a^Lom-Lclay	$y=-\,0.75\ast pH-2.25\ast OM-5.88\ast V+0.00\ast Clay-6.99$	4.00	100.00
Lclay	$y=0.53\ast pH-1.19\ast OM-1.32\ast V-1.27\ast Clay-2.03$	10.00	100.00
Hom-Hclay	$y=-\,0.65\ast pH+3.61\ast OM-2.89\ast V+0.83\ast Clay-5.40$	5.00	100.00
HpH-Hclay	$y=0.12\ast pH+0.36\ast OM+6.40\ast V+1.08\ast Clay-5.93$	8.00	100.00

^a^Groups obtained by *cluster* analysis. ^b^Number of soil samples that make up each group previously defined by cluster analysis. ^c^Percentage of hit of the samples classified in each group, according to the discriminant analysis.

### Equilibrium time, sorption and desorption in the different soil groups

**Figure 2:**
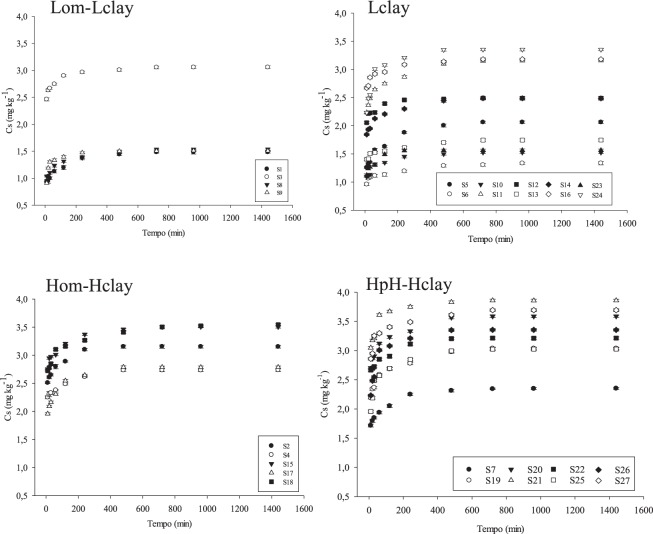
Kinetic curve of diuron sorption in the four soil groups (Lom-Lclay, Lclay, Hom-Hclay and HpH-Hclay). The bars correspond to the standard error of the mean.

After the multivariate analysis, new interpretations could be derived from the equilibrium time, sorption, and desorption into each soil group. The time necessary for the concentration in the solution to become constant differed among the groups Lom-Lclay, Lclay, Hom-Hclay, and Hph-Hclay. Soils belonging to groups Hom-Hclay and Hph-Hclay showed an equilibrium time of 8 hours (Fig. 2). However, for Lom-Lclay and Lclay, the equilibrium time was 4 hours (Fig. 2). The reduced equilibrium time for Lom-Lclay might be related to the lower OM and clay content of their soils (Table 4).

**Table 4:** Average and standard deviation of the sorption coefficient, desorption coefficient and physical and chemical attributes for the soil groups formed from Ward’s cluster analysis.

Groups	pH (H_2_O)	^a^OM	^b^ *V*	Clay	Kfs	Kfd	Contamination potential
			%				
Lom-Lclay	4.7 (±0.1)	0.9 (±0.2)	27.0 (±4.1)	26.8 (±6.7)	5.9 (±1.2)	10.9 (±0.6)	High risk
Lclay	5.9 (±0.3)	1.3 (±0.2)	44.4 (±3.1)	17.1 (±3.6)	7.5 (±1.1)	10.4 (±1.3)	High risk
Hom-Hclay	5.3 (±0.6)	2.8 (±0.3)	39.2 (±4.4)	45.2 (±4.9)	11.2 (±1.2)	13.8 (±1.2)	Low risk
HpH-Hclay	6.3 (±0.4)	1.9 (±0.3)	74.0 (±4.7)	56.3 (±7.6)	10.1 (±1.1)	11.5 (±1.4)	Low risk

The averages of the physical-chemical attributes were obtained from the data in Tables S2 and S3. ^a^Organic matter. ^b^Base saturation.

**Figure 3:**
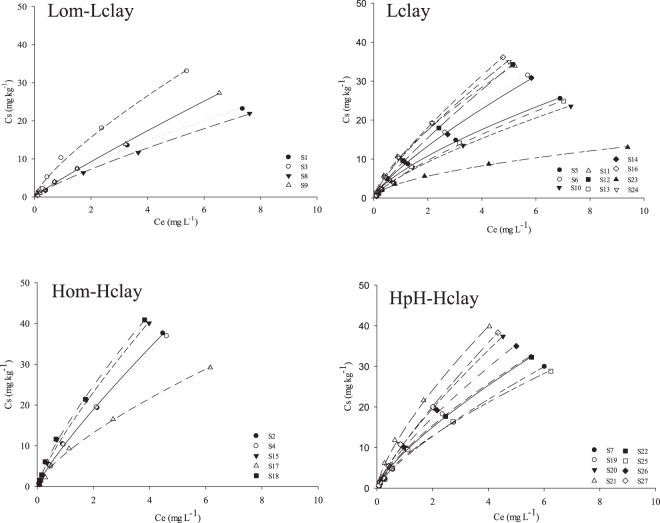
Freundlich isotherms of diuron sorption in the four soil groups (Lom-Lclay, Lclay, Hom-Hclay and HpH-Hclay).

The main characteristics of the soil groupings in Hom-Hclay and HpH-Hclay were the highest average for OM and clay (Table 4). These attributes have many blind sites that are able to sorb the diuron, extending the time to reach equilibrium. The Freundlich sorption isotherms of the soils of Lom-Lclay, Lclay, Hom-Hclay, and HpH-Hclay are presented in Fig. 3. The Hom-Hclay and HpH-Hclay groups demonstrated the highest mean value for Kfs (Table 4). Additionally, a higher OM content (2.8 ± 0.26) was verified in soils belonging to the HOM-HCLAY, as well as the second highest clay content (45.2 ± 4.9) (Table 4). In HpH-Hclay, the OM was 1.8 ± 0.27, and the clay content was close to Hom-Hclay (45.2 ± 4.9). In addition to the longer equilibrium time, a greater number of blind sites present in the OM and clay provide a higher sorption capacity for soils of Hom-Hclay and HpH-Hclay (11,2 ± 1.1 and 10.1 ± 1.2, respectively, Table 4).

The mean value for pH in HpH-Hclay (6.3 ± 0.35) was higher than in Hom-Hclay (5.3 ± 0.56). Despite the lower amount of OM and higher pH for HPH-HCLAY compared with Hom-Hclay, which reduces the sorption of diuron, the Kf_s_ values between these groups were similar. In addition to these attributes, the main observed difference between these groups was the number of cations composing the *V* (HpH-Hclay with 74.0 ± 4.7 and Hom-Hclay with 39.2 ± 4.4, Table 4).

**Figure 4:**
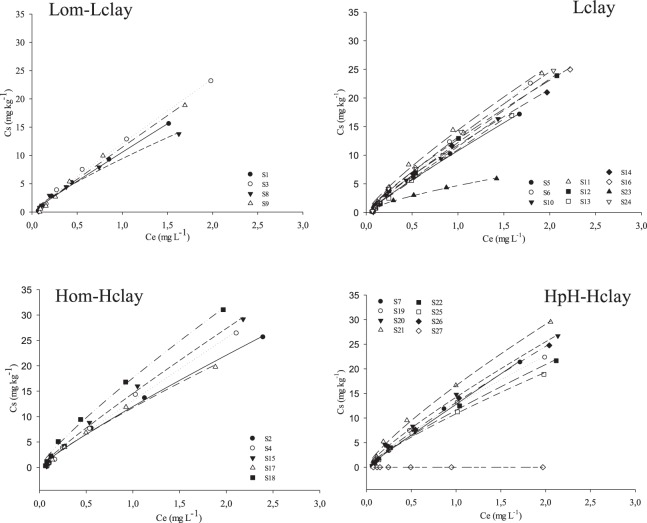
Freundlich’s isotherms of diuron desorption in the four soil groups (Lom-Lclay, Lclay, Lclay and HpH-Hclay).

The Lom-Lclay and Lclay presented the lowest Kfs (5.9 ± 1.2 and 7.5 ± 1.1) in comparison to the other groups. The soils that were separated in this group had the lowest values for OM and *V* and the second lowest value for clay (26.8 ± 6.7). The clay content was lowest in Lclay (17.1 ± 3.6) (Table 4), evidencing the importance of the attributes OM and clay in the sorption of diuron and indicating that soil with a low value for these proprieties has a lower capacity to adsorb this herbicide. The Freundlich isotherms for desorption of the four groups are shown in Fig. 4. The order for the Kfd values was Hom-Hclay > HpH-Hclay > Lclay > Lom-Lclay (Table 4). In general, an inverse relationship was observed between sorption and desorption for all the soil groups, among which those with high sorption displayed a lower desorption of diuron.

Analysis of the results found by grouping allowed verification that the soils within each group had a similar sorption and desorption capacity for the diuron. However, it will be essential to test the generalization ability of the cluster to other soils that were not used in the present analysis. For this purpose, a discriminant analysis was performed, and the obtained functions were used to classify 18 soils with information obtained from the literature. When extrapolated to the other 18 soils, the discriminant function allowed their classification within the four groups (Table 5). The linear function provided by the AD separated the other 18 soils into four groups (Table 5). Among these 18 soils, a greater number of soils (11) were classified into Lom-Lclay and Lclay (Table 5). The soils with low sorption were classified into Lom-Lclay or Lclay, and those with high sorption were classified into Hom-Hclay or HpH-Hclay (Table 5).

**Table 5:** Grouping another 18 soils into of the four groups created in the cluster analysis by the classification functions based on the attributes pH, OM, *V*, and clay content.

Soils	^a^Kfs	^b^Kfd	Scores values				Classification
			Lom-Lclay	Lclay	Hom-Hclay	HpH-Hclay	
^c^S_E_7	1.8	1.7	0.5	0.5	−5.4	−12.8	1.0
S_E_8	2.3	1.3	1.8	1.6	−7.3	−13.7	
S_E_9	0.8	1.6	2.9	1.5	−7.4	−14.1	
S_E_10	2.1	1.3	3.0	1.1	−6.4	−14.3	
S_E_11	1.8	1.8	−3.1	0.4	−5.6	−10.8	2.0
S_E_12	3.3	1.5	−3.6	−0.5	−4.3	−10.2	
S_E_13	2.7	1.5	−5.5	−0.8	−3.5	−9.4	
S_E_15	4.6	9.9	−7.0	−0.8	−5.5	−7.4	
S_E_16	2.2	16.1	−7.4	−1.0	−5.4	−7.0	
S_E_17	3.7	9.5	−6.5	−1.1	−5.9	−7.0	
S_E_14	3.3	11.7	−2.5	1.4	−6.3	−11.9	
S_E_5	7.8	0.1	−12.2	−4.6	−12.4	4.2	3.0
S_E_1	5.7	0.1	−11.5	−5.8	−6.7	1.9	4.0
S_E_2	6.2	1.2	−15.8	−6.9	−4.7	4.1	
S_E_3	8.6	2.5	−13.2	−6.5	−2.2	0.7	
S_E_4	11.2	1.4	−23.8	−8.1	−4.2	9.4	
S_E_6	18.0	6.7	−18.7	−6.9	−0.2	2.7	

^a^Sorption coefficient. ^b^Desorption coefficient. ^c^Soils obtained from the others studies^25–29^.

### Leaching potential of diuron in brazilian states

**Figure 5:**
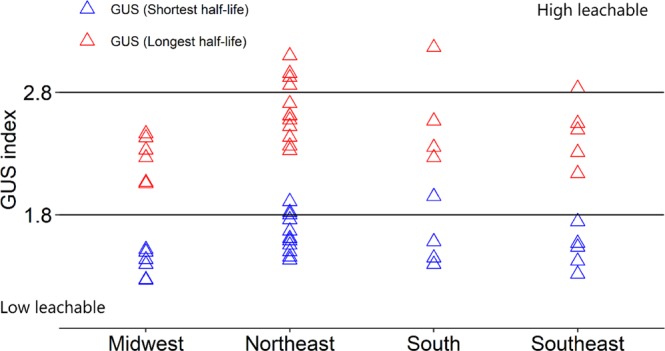
Leaching risk of diuron in 27 Brazilian soils according to the GUS index for soil half-life of 15 and 150 days.

Values for the GUS index below 1.8 indicated that the soil presented a low risk for leaching of the herbicide. Conversely, values above 2.8 were found for soils with a high risk of leaching. Thus, the leaching potential of diuron considering a half-life equivalent to 15 days was moderate and not leachable for all Brazilian states (Fig. 5). However, if we consider a half-life of 150 days for diuron, soils collected in the states in the northeast, south, and southeast presented a high risk for leaching of this herbicide (Fig. 5). Among the soils with a high risk of diuron leaching, a higher frequency of soils collected in the northeast was observed in comparison to the other Brazilian states (Fig. 5). These soils sampled in the northeast were classified in Lom-Lclay (single group with soils of a state), a group with lower sorption values for diuron (Table 4). The Lom-Lclay group presented the lowest mean value of OM (28, 50, and 77% lower) compared with the other groups, and this difference might be responsible for the lower sorption and higher risk of herbicide leaching.

## Discussion

The physical and chemical properties of soils define the associated behavior of herbicides. Among these attributes, OM, clay, and pH have been widely studied, and their importance in the sorption and desorption of herbicides has been previously reported for both ionic and nonionic molecules^30,31^. Therefore, all others attribute that showed a significant correlation, positive or negative, with OM, clay, and pH were omitted from further analyses (CA, FA, and DA). This procedure is essential in multivariate analysis to avoid redundancy in the results due to the use of several variables capable of explaining the same phenomenon^32,33^.

In addition to the abovementioned variables, *V* correlated significantly with the attributes Ca^2+^, Mg^2+^, H + AL, and CEC. Statistically, through the use of *V*, it is possible to discard the other attributes, reducing the number of variables in subsequent analyses. However, it is necessary to choose variables that have some role in the sorption and desorption of diuron. The saturation of bases has not been studied as an attribute that affects the behavior of herbicides in soil. However, this variable indirectly encompasses the importance of CEC and cations of the basic reaction in the sorption and desorption of herbicides. Studies have previously discussed how CEC and cations can participate in these processes, such as glyphosate^34^, atrazine^35^ and 2,4-D^36^. Therefore, the choice of *V* was considered more appropriate in this work for the AC, AF, and AD analyses.

The FA of the attributes pH, OM, *V*, and clay considering three main components (Factorial 1, Factorial 2 and Factorial 3) showed that these variables explained 94% of the current variability in the 27 solos. For CA, it was essential that the variables could detect the differences and similarities of the soils. Therefore, the pH, OM, *V*, and clay would probably be able to group the soils into homogeneous groups, even without considering the other attributes.

The *V* and clay attributes were more important for Factorial 1, which detected 49.66% of the variance among the soils. Some factors can explain these findings. For example, the content and type of clay of the soil is a result of the degree of weathering and the rock origin^37^. Therefore, due to the diversity of rocks^38^ and climatic conditions^39^ in Brazil, a high variability of the clay contents constituting the soil is expected, as shown in the AF for F1. The high accumulated variance for base saturation is directly related to the variability in clay content, since clay is the primary attribute responsible for the retention of cations in the soil, such as Ca^+2^, Mg^+2^, K^+^, and Na^+^ ^40,41^.

For Factorial 2, pH and OM were the variables with higher importance and contributed to 27.51% of the total variance among soils. Despite the high diversity of Brazilian soils concerning their chemical and physical attributes, the values of pH and OM demonstrated less variation compared with clay^21,33^. This phenomenon resulted from the high degree of weathering of Brazilian soils due to favorable climatic conditions. The greater homogeneity of pH and MO among the 27 soils explains the lower contribution of these attributes to the total accumulated variance. The great total variance detected using only the attributes pH, OM, *V* and clay was considered satisfactory, and therefore these soil properties were used in the cluster analysis with good results for group differentiation.

The number of soil samples for each group depended on the number of soils with similar characteristics for the attributes pH, OM, *V* and clay, as well as on the distance used to separate the groups. Although some authors have reported criteria for the ideal Euclidean distance, many studies consider this distance subjectively^20,21,32,33^. In this work, the Euclidean distance equal to 5.8 separated the soils into four groups. The increase in the Euclidean distance reduced the number of groups formed. However, there is a risk of grouping soils with different characteristics^32^, which may represent a problem for the study of herbicide behavior in soils, since soils with different Kfs and Kfd for diuron may be included in the same group. A technique to validate the appropriate grouping of soils is the discriminant analysis^32,33^. The discriminant analysis showed that the classification functions were able to group the 27 soils with 100% accuracy. This result is a reflection of the excellent grouping of soils through the selected variables and Euclidean distance.

After the soil selection and grouping stages, interpretation of the equilibrium time, sorption and desorption of soils within each group showed that the attributes pH, OM, *V*, and clay had different influences on the diuron behavior in the groups (Lom-Lclay, Lclay, Hom-Hclay, HpH-Hclay). Evaluation of the Kfs and Kfd values for each formed group allowed us to confirm the similar patterns for the attributes pH, OM, *V*, and clay for the equilibrium time and the sorption and desorption coefficients.

The adsorption of an herbicide to soil usually occurs in three phases^42^. For diuron, these behaviors were observed for all groups, but the time to reach the last phase (equilibrium between the soil and solution concentrations) occurred at a faster rate in Lom-Lclay and Lclay compared with Hom-Hclay and HpH-Hclay. The initiation of diuron sorption in the soil occurred quickly due to the higher presence of sites capable of interacting with the herbicide^42^. However, as the herbicide occupied the binding sites, sorption became slower due to site saturation, resulting in equilibrium between the adsorbed concentration and the solution^43^. Soils of Lom-Lclay and Lclay have few sites available to adsorb diuron, accelerating saturation. In contrast to the Lom-Lclay and Lclay soils, the higher availability of sites on organic and mineral colloid surfaces of the soils grouped into Hom-Hclay and HpH-Hclay allowed greater adsorption of the diuron. Thus, greater amounts of the herbicide and time were necessary to reach saturation compared with Lom-Lclay and Lclay soils^42^.

Soils of Lom-Lclay and Lclay presented a lower sorption and higher desorption for diuron. In contrast, the soils that were grouped in Hom-Hclay and HpH-Hclay were able to sorb more diuron, with lower desorption. The highest sorption and lowest desorption of diuron in Hom-Hclay and HpH-Hclay compared with Lom-Lclay and Lclay resulted from the higher mean value of OM (2.8 ± 0.26). Among the constituents of the soil capable of adsorbing diuron, OM is the most important property in determining the sorption capacity of diuron^5,44,45^. The presence of the urea grouping and an aromatic ring without steric hindrance in the diuron molecule allows the development of hydrogen bonds^9^ and van der Waals forces^46^ in carboxylic and phenolic sites and aromatics of OM. This combination between herbicide and OM properties increased diuron sorption in soils in the Hom-Hclay and HpH-Hclay groups.

Another attribute utilized in the AC was the soil pH. Diuron is a neutral herbicide at pH 6.0 and can receive protons (H^+^) in soils with a pH below that value^47^. The higher pH observed in HpH-Hclay compared with the others groups could reduce diuron sorption. This herbicide can become a neutral molecule, reducing the force of Coulomb attractions with the positive charges of the soil. This phenomenon reduces the sorption of diuron and, consequently, the Kfs coefficient. However, the Kfs in HpH-Hclay was high compared with the other groups with lower values for soil pH. This finding seems contradictory, as other soil attributes should be more important to adsorb diuron.

An attribute that is probably responsible for the high sorption of diuron in soils of HpH-Hclay is *V*. In this group, *V* as well as Kfs were high. Some studies have evaluated the effect of bivalent cations, such as Ca^+2^, Zn^+2^, Ni^+2^, Cu^+2^, and Co^+2^, in the adsorption of herbicides^1,9,34–36^. An initial observed effect is the surface complex. These cations can form complexes with these herbicides and increase the adsorption of these herbicides with the clay surfaces, elevating the sorption capacity of the soils^20,32^. In addition, bivalent cations may interact with herbicides forming cation-herbicide complexes, or cation-herbicide-cation, with high stability^43,44^, and these complexes may precipitate in the soil solution, increasing the sorption of the herbicide. Diuron has two chlorine atoms attached to an aromatic ring. This condition allows the formation of a site with higher electronegativity capable of attracting cations. Thus, in soils with a high concentration of cations, as in HpH-Hclay, diuron may be more strongly adsorbed due to the abovementioned processes.

The high Kfd observed in Hom-Hclay and HpH-Hclay indicates a lower desorption of the herbicide, requiring higher energy to break the herbicide-soil bond^8^. Therefore, in soils grouped in Hom-Hclay and HpH-Hclay, there is a lower chance that the herbicide will return to the soil solution. As mentioned previously, the contents of MO were higher in Hom-Hclay and HpH-Hclay than in the other groups, as well as the number of interactions established between the soil and diuron. This mechanism increases both the sorption and stability of diuron-soil bonds^8^.

Soils with similar properties to Lom-Lclay and Lclay may present a high risk of groundwater and surface water contamination due to the low sorption capacity of diuron. When diuron is not adsorbed to soil, it may be available in the soil solution. Consequently, in regions with a higher rainfall intensity, diuron may be transported by leaching or surface runoff, contaminating rivers and groundwater^44^. However, the Hom-Hclay and HpH-Hclay groups showed high sorption and low desorption of diuron; thereby, the risk of contamination of surface and groundwater is low for this group. These results confirm the second hypothesis of this study. The soils were grouped according to their physical-chemical characteristics, and each group presented a pattern of diuron behavior in the soil, in this case, two groups with low sorption and high desorption soils, and two groups with high sorption capacity and low desorption.

The soils in Lom-Lclay were all from the Brazilian northeast, more specifically the state of Rio Grande do Norte. This grouping resulted from the similarity of the soils in this state^33^. The main characteristic of the soils in this state is the low OM and clay content, as well as the high pH. These associated factors resulted in a higher risk for diuron leaching according to the GUS index, especially when considering the higher herbicide half-life conditions. Only a few soils from other regions of Brazil (south, southeast, and central) showed a higher risk of diuron leaching. Therefore, for the northeastern sugarcane regions, greater precautions regarding the application of diuron are necessary, confirming our third hypothesis.

In the correlation analyses, FA, CA and DA were able to separate the 27 soils into groups with a similar sorption and desorption pattern. This last analysis (DA) assessed the effectiveness of the grouping and provided weights for the variables of the classification functions for each group. These functions were used to separate the other 18 soils with previously measured sorption and desorption coefficients for diuron, thus addressing the four hypothesis. The classification functions separated the 18 soils based on the similarity of the pH, OM, *V*, and clay attributes. After grouping, the sorption and desorption coefficients were observed to confirm whether the soils with low sorption and high desorption belonged to the Lom-Lclay or Lclay group, and those with high sorption and low desorption to the Hom-Hclay or HpH-Hclay group. This last step also permitted evaluation of the generalization ability of the functions^32,33^. According to the results, the 11 soils included in the Lom-Lclay or Lclay groups presented low sorption for diuron, whereas the others included in Hom-Hclay or HpH-Hclay demonstrated high sorption for this herbicide. This finding is extremely encouraging because it confirms that other soils, even if they belong to other countries, can be separated into groups with a similar pattern for diuron adsorption based only on their pH, OM, *V* and clay attributes.

Multivariate statistical analysis is a tool that is capable of providing new interpretations concerning herbicide behavior in soil. For example, no study has reported the greatest risk for contamination of groundwater in regions of the northeast of Brazilian, which was elucidated in the present analysis. Despite the significant findings in this work, the accuracy and precision of the analysis can be substantially improved to utilize a larger database. Thus, application of this method considering a larger number of samples could increase the correlation between the grouping and behavior of diuron, generating broader and more accurate values to estimate the behavior of herbicides in different soils. Analyses generated from a greater amount of data could optimize the grouping of soils, providing linear functions that allow identification of the risk of contamination by diuron in any region using only the physicochemical characteristics of the soil.

## Conclusions

The multivariate analysis revealed significant diuron-related changes in the soil composition of different Brazilian regions. This technique can be applied to determine groups of soils with a similar sorption and desorption capacity for diuron based on the physical and chemical attributes of the soils. The pH, organic matter and clay content, as well as the base saturation are the attributes with the greatest influence on the sorption and desorption of diuron and provide good grouping of the soils. These proprieties can be used in the determination of homogeneous soil groups for these features. Soils with Kfs values of 5.9 to 7.5 have a medium-high potential for leaching under conditions of a higher half-life of diuron. Among the evaluated states, Rio Grande do Norte demonstrated the highest contamination risk by diuron when the herbicide is applied in sugarcane plantations. The classification functions considering the attributes pH, organic matter content and clay, as well as base saturation can be applied to classify other soils with a high or low capacity to adsorb diuron, demonstrating good generalization ability.

### Main finding of the work

The multivariate analysis provided deeper insight into the behavior of diuron in twenty-seven soils collected in different regions of Brazil. Soils that present similar quantitative characteristics regarding physical-chemical attributes display the same sorption and desorption pattern of diuron, independent of the sampled region. Diuron shows higher leaching potential according to the GUS index for soils collected from agricultural areas of Northeast Brazil. For the other regions of the country, there is a reduced risk of diuron leaching.

## Supplementary information

Supplementary file
