# Supplementary material for: Multivariate analysis reveals significant diuron-related changes in the soil composition of different Brazilian regions
Source: Sci Rep. 2019 May 27;9:7900. doi: 10.1038/s41598-019-44405-x (PMC6536495; doi:10.1038/s41598-019-44405-x)
Supplement: Supplementary file 1 — Supplementary file [file 41598_2019_44405_MOESM1_ESM.docx]

**Multivariate analysis reveals significant diuron-related changes in the soil composition of different Brazilian regions**

Paulo Sergio Fernandes das Chagas^a^, Matheus de Freitas Souza^a^, Jeferson Luiz Dallabona Dombroski^a^, Rubem Silvério De Oliveira Junior^b^, Glauber Henrique De Sousa Nunes^a^, Gustavo Antônio Mendes Pereira^c^, Tatiane Severo Silva^a,*^, Daniel Valadão Silva^a^

**^a^Universidade Federal Rural do Semi-Árido, Mossoró, Rio Grande do Norte, Brazil.**

**^b^Universidade Estadual de Maringá, Maringá, Paraná, Brazil.**

**^c^Universidade Federal de Viçosa, Viçosa, Minas Gerais, Brazil.**

***Corresponding author:** Tatiane Severo Silva.

*E-mail address:* [tatiane.severosilva@gmail.com](mailto:tatiane.severosilva@gmail.com). Av. Francisco Mota, 572, Costa e Silva, Ramal: 1948, Caixa-postal: 137, Fone: (84) 33178548, CEP: 59625-900, Mossoró, Rio Grande do Norte, Brazil.


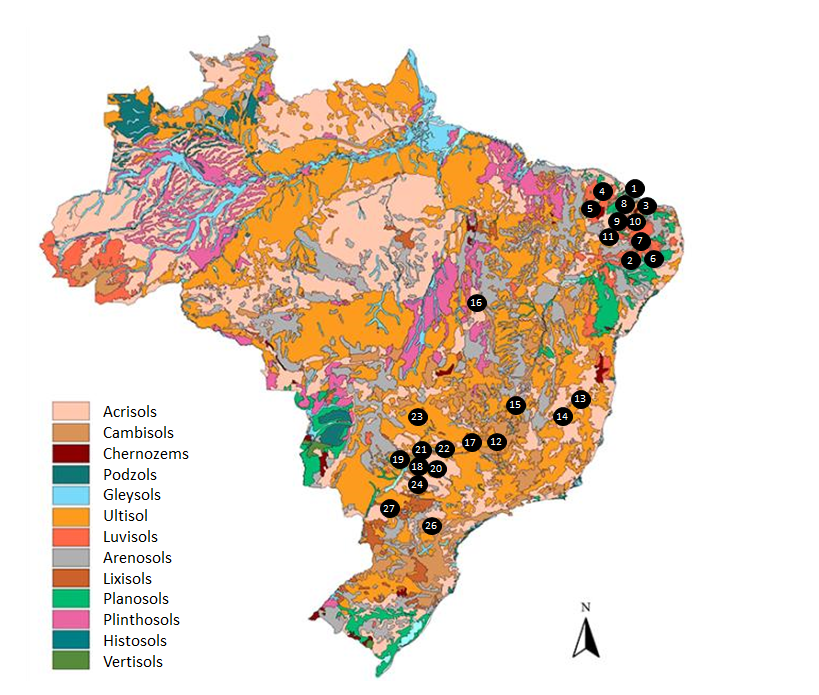


**Figure S1.** Collection sites of soil samples. Adapted from Embrapa: <https://www.infoteca.cnptia.embrapa.br/handle/doc/920267>


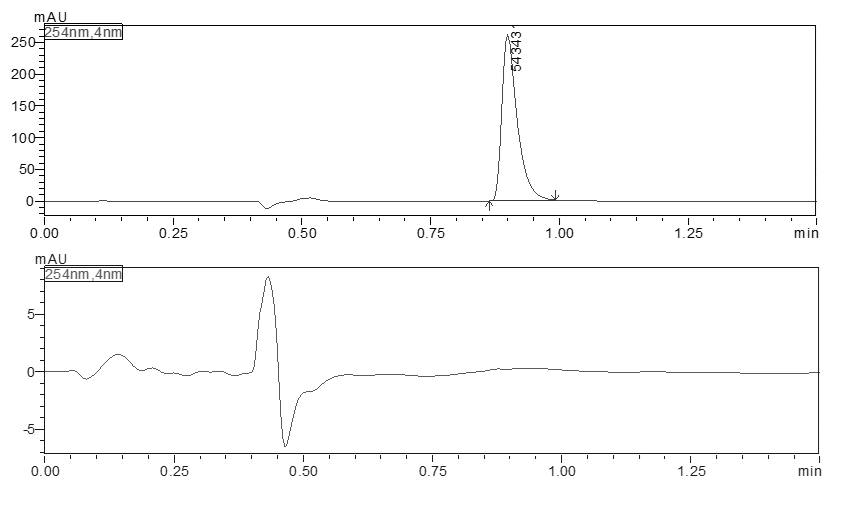


a

b

**Figure S2.** Chromatogram generated from fortified samples with a known concentration of diuron (a). Chromatogram generated from samples without diuron (b), demonstrating the absence of the herbicide in the soil samples. Peak absorption of diuron 0.9 min.


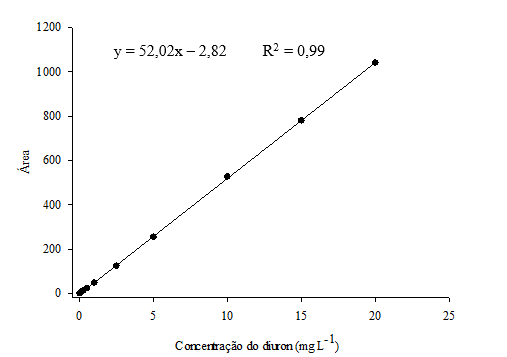


**Figure S3.** The linearity of the method at increasing concentrations of diuron.

| State | City | Soil | Coordinates |
| --- | --- | --- | --- |
| Rio Grande do Norte | Tibau | S1 | 4° 38' 00" S e 37° 15' 00" O |
|  | Baía Formosa | S3 | 6° 22' 00" S e 35° 00' 00" O |
|  | Mossoró | S8 | 5° 3' 39.8" S e 37° 23' 44.6" O |
|  | Mossoró | S9 | 5° 3' 37.7" S e 37° 24' 14.4" O |
|  | Mossoró | S10 | 5° 3' 58.5" S e 37° 24' 3.2" O |
|  | Pedro Avelino | S11 | 6° 25' 48" S e 35° 13' 28" O |
| Ceará | Quixeré | S7 | 5° 4' 44" S e 37° 48' 3" O |
| Alagoas | Maceió | S2 | 9° 30' 25" S e 35° 39' 25" O |
|  | Maceió | S6 | 9° 39' 21" S e 35° 44' 38" O |
| Pernambuco | Carpina | S4 | 7° 50' 35" S e 35° 16' 21" O |
|  | Carpina | S5 | 7° 48' 18" S e 35° 17' 38" O |
| São Paulo | Taquaritinga | S12 | 21° 24' 36" S e 48° 30' 36" O |
|  | Paulínia | S17 | 22° 54' 36" S e 47° 48' 12" O |
| Minas Gerais | Santa Vitória | S13 | 18° 51' 00" S e 50° 7' 12" O |
|  | Viçosa | S14 | 20° 42' 36" S e 42° 49' 48" O |
|  | Uberlândia | S15 | 18° 48' 36" S e 48° 25' 12" O |
| Tocantins | Gurupi | S16 | 10° 45' 00" S e 49° 45' 12" O |
| Mato Grosso do Sul | Rio Brilhante | S18 | 21° 54' 19" S e 54° 31' 50" O |
|  | Amambai | S19 | 23° 04' 44" S e 55° 08' 27" O |
|  | Dourados | S20 | 22° 12' 02" S e 54° 39' 32" O |
|  | Aral Moreira | S21 | 22° 58' 21" S e 55° 35' 00" O |
|  | Laguna Carapã | S22 | 22° 34' 46" S e 55° 09' 59" O |
|  | Caarapó | S23 | 23° 39' 23" S e 54° 49' 22" O |
| Paraná | Maringá | S24 | 23° 21' 07" S e 52° 04' 09" O |
|  | Iguatemi | S25 | 23° 23' 44" S e 51° 57' 04" O |
|  | Sarandi | S26 | 23° 24' 21" S e 51° 49' 55" O |
|  | Paranavaí | S27 | 23° 06' 22" S e 52° 30' 25" O |

**Table S1.** Collection sites of soil samples

| SOIL | pH | MO | P | K | Ca^+2^ | Mg^+2^ | Al^+3^ | H+Al | CTC | v | m |
| --- | --- | --- | --- | --- | --- | --- | --- | --- | --- | --- | --- |
|  | (water) | (dag/kg) | (mg/dm^3^) | | ---------------(cmolc/dm^3^)------------- | | | | | (%) | |
| S1 | 4.8 | 1.0 | 0.5 | 58.9 | 0.9 | 0.2 | 0.2 | 4.2 | 5.9 | 28.0 | 11.0 |
| S2 | 4.5 | 3.4 | 35.6 | 180.6 | 2.8 | 0.7 | 0.3 | 9.0 | 13.1 | 34.0 | 6.0 |
| S3 | 4.8 | 1.4 | 6.7 | 30.0 | 0.4 | 0.6 | 0.2 | 8.5 | 10.1 | 16.0 | 11.0 |
| S4 | 4.6 | 2.5 | 0.7 | 34.0 | 1.5 | 2.8 | 0.5 | 11.8 | 16.8 | 30.0 | 9.0 |
| S5 | 5.8 | 1.2 | 4.7 | 108.3 | 1.8 | 1.7 | 0.0 | 4.7 | 1.7 | 46.7 | 0.0 |
| S6 | 5.8 | 1.7 | 0.9 | 108.1 | 1.3 | 1.7 | 0.0 | 5.4 | 1.7 | 40.6 | 0.0 |
| S7 | 6.5 | 1.5 | 2.2 | 97.0 | 5.4 | 2.9 | 0.0 | 4.9 | 2.9 | 64.5 | 0.0 |
| S8 | 4.4 | 0.5 | 0.1 | 21.9 | 0.5 | 1.3 | 0.4 | 4.1 | 6.4 | 36.0 | 15.0 |
| S9 | 4.5 | 0.7 | 0.2 | 28.0 | 0.8 | 0.8 | 3.3 | 5.4 | 7.6 | 28.0 | 60.0 |
| S10 | 5.0 | 1.3 | 1.7 | 64.1 | 3.5 | 0.6 | 0.5 | 5.7 | 10.7 | 45.0 | 10.0 |
| S11 | 5.5 | 0.7 | 2.6 | 108.3 | 1.4 | 1.2 | 0.9 | 3.9 | 2.1 | 45.5 | 21.4 |
| S12 | 6.4 | 1.2 | 4.1 | 90.3 | 3.5 | 1.5 | 0.0 | 6.2 | 1.5 | 47.5 | 0.0 |
| S13 | 6.6 | 1.5 | 2.9 | 92.6 | 2.0 | 1.3 | 0.0 | 5.2 | 1.3 | 41.9 | 0.0 |
| S14 | 6.6 | 1.0 | 4.4 | 94.8 | 1.2 | 0.4 | 0.0 | 4.9 | 0.4 | 30.4 | 0.0 |
| S15 | 6.9 | 3.1 | 2.9 | 97.0 | 4.2 | 1.1 | 0.0 | 6.6 | 1.1 | 47.0 | 0.0 |
| S16 | 6.1 | 2.0 | 9.5 | 97.0 | 3.2 | 2.2 | 0.0 | 7.5 | 2.2 | 44.5 | 0.0 |
| S17 | 5.8 | 2.7 | 7.3 | 114.7 | 3.1 | 0.8 | 0.3 | 8.9 | 1.1 | 35.0 | 5.7 |
| S18 | 4.9 | 2.1 | 15.7 | 222.3 | 4.9 | 1.7 | 0.2 | 7.3 | 7.5 | 49.7 | 3.2 |
| S19 | 7.2 | 1.1 | 149.0 | 195.0 | 10.7 | 0.7 | 0.0 | 2.2 | 11.5 | 83.8 | 0.0 |
| S20 | 5.2 | 1.9 | 62.8 | 483.6 | 8.6 | 1.8 | 0.1 | 6.9 | 11.7 | 62.6 | 0.6 |
| S21 | 6.5 | 2.9 | 19.6 | 390.0 | 8.5 | 4.0 | 0.0 | 3.5 | 13.9 | 79.4 | 0.0 |
| S22 | 6.7 | 1.9 | 118.9 | 327.6 | 9.0 | 5.9 | 0.0 | 2.8 | 15.8 | 84.5 | 0.0 |
| S23 | 5.5 | 1.6 | 5.4 | 109.0 | 3.4 | 1.7 | 0.0 | 3.8 | 5.4 | 58.4 | 0.3 |
| S24 | 5.3 | 0.4 | 14.2 | 25.1 | 1.9 | 0.5 | 0.1 | 3.4 | 2.6 | 42.7 | 5.8 |
| S25 | 5.2 | 1.4 | 0.1 | 230.1 | 6.8 | 2.7 | 0.1 | 5.3 | 10.3 | 65.7 | 0.6 |
| S26 | 6.4 | 2.1 | 2.3 | 483.6 | 12.6 | 2.5 | 0.0 | 3.5 | 16.6 | 82.3 | 0.0 |
| S27 | 6.3 | 1.8 | 16.6 | 105.3 | 4.1 | 1.2 | 0.0 | 2.5 | 5.6 | 69.1 | 0.0 |

**Table S2.** Chemical characteristics of soil samples

*Analyzes were performed according to methodology proposed by the Brazilian Agricultural Research Company EMBRAPA (2013). Laboratory of Soil Analysis of UFERSA.

| Soil | Sand | Silt | Clay |
| --- | --- | --- | --- |
|  | % | | |
| S1 | 67.7 | 0.6 | 31.7 |
| S2 | 50.0 | 28.0 | 22.0 |
| S3 | 85.2 | 2.3 | 12.4 |
| S4 | 49.0 | 8.0 | 43.0 |
| S5 | 72.0 | 14.0 | 14.0 |
| S6 | 63.0 | 11.0 | 23.0 |
| S7 | 48.0 | 14.0 | 37.0 |
| S8 | 21.0 | 20.0 | 43.0 |
| S9 | 77.6 | 2.4 | 20.0 |
| S10 | 86.7 | 4.3 | 9.0 |
| S11 | 93.0 | 5.0 | 2.0 |
| S12 | 77.5 | 4.5 | 18.0 |
| S13 | 81.0 | 4.0 | 15.0 |
| S14 | 68.1 | 9.9 | 22.0 |
| S15 | 48.3 | 0.1 | 51.6 |
| S16 | 41.5 | 16.3 | 42.2 |
| S17 | 36.5 | 11.3 | 52.2 |
| S18 | 38.0 | 4.9 | 57.1 |
| S19 | 53.8 | 1.2 | 45.0 |
| S20 | 21.1 | 5.3 | 76.3 |
| S21 | 13.6 | 8.8 | 77.6 |
| S22 | 45.3 | 9.5 | 45.2 |
| S23 | 72.3 | 3.2 | 24.5 |
| S24 | 78.1 | 1.2 | 20.7 |
| S25 | 8.3 | 8.5 | 83.2 |
| S26 | 15.5 | 11.1 | 73.4 |
| S27 | 85.0 | 2.6 | 12.4 |

**Table S3**. Physical characteristics of soil samples

*Analyzes were performed according to methodology proposed by the Brazilian Agricultural Research Company EMBRAPA (2013). UFERSA Soil Analysis Laboratory.
